# Novel Approaches to Minimizing Mycotoxin Contamination

**DOI:** 10.3390/toxins12040216

**Published:** 2020-03-29

**Authors:** Mar Rodríguez, Félix Núñez

**Affiliations:** Food Hygiene and Safety, Meat and Meat Products Research Institute, Faculty of Veterinary Science, University of Extremadura, Avda. de las Ciencias, s/n, 10003 Cáceres, Spain

Contamination of foods and agricultural commodities by various types of toxigenic fungi is a concerning issue for human and animal health. Molds naturally present in foods can produce mycotoxins and contaminate foodstuffs under favorable conditions of temperature, relative humidity, pH, and nutrient availability. Mycotoxins are, in general, stable molecules, which are difficult to remove from foods once they have been produced. Therefore, the prevention of mycotoxin contamination is one of the main goals of the agriculture and food industries.

Chemical control or decontamination techniques may be quite efficient. However, the more sustainable and restricted use of fungicides, the lack of efficiency in some foods, and the consumer demand for chemical residue-free foods require new approaches to control this hazard. Therefore, food safety demands permanent research efforts for exploring new strategies to reduce mycotoxin contamination.

This Special Issue contains original contributions and reviews that advance the knowledge about the most current promising approaches to minimize mycotoxin contamination, including biological control agents (BCAs), phytochemical antifungal compounds, enzyme detoxification, and the use of novel technologies. Most of the studies focus on *Fusarium* toxins, but the toxicity of aflatoxins and ochratoxin A is also addressed. In addition, a few studies are focused on the control of plant pathogenic fungi.

Several studies examined the potential of biological control strategies as alternative methods in both plant pathogenic and mycotoxigenic fungi. Within the group of articles about biological control strategies, Ntushelo et al. [1] reported that *Bacillus* species adopt various mechanisms, including the production of bioactive compounds, to inhibit the growth of the mycotoxin-producing plant pathogenic fungus *Fusarium graminearium* and provided a perspective of the techniques used to study antagonist metabolites. Moraes-Bazioli et al. [2] review the potential of BCAs, including bacteria, yeasts, and natural plant products for the control of *Penicillium digitatum*, *Penicillium italicum*, and *Geotrichum citriaurantii*, which are responsible for postharvest citrus diseases. Zearalenone (ZEA) is an estrogenic mycotoxin which can cause loss in animal production. Chen et al. [3] selected a *Bacillus* strain with a strong esterase activity that exhibited a high ZEA detoxification capability in maize using a fermentation process to validate their potential application in the feed industry. In the same sense, Liu et al. [4] tested the combination of probiotic strains from *Bacillus subtilis* and *Candida utilis* with cell-free extracts from *Aspergillus oryzae* to degrade ZEA. Interestingly, the authors showed the BCA effect of alleviating the negative impact of ZEA on normal growth performance in pig keeping. Aflatoxin M1 (AFM1) is secreted in the milk of lactating mammals through the ingestion of feedstuff contaminated by aflatoxin B1, being a health concern for dairy industries and consumers of dairy products. Assaf et al. [5] review AFM1 decontamination methods including different bio-adsorbents such as bacteria, yeasts, or mixtures of both. The efficiency of these decontamination methods in addition to their plausible experimental variants, advantages, limitations, and prospective applications are broadly discussed. Ochratoxin A (OTA) is the mycotoxin most commonly found in meat products, which contribute significantly to human exposure to this toxin. Cebrian et al. [6] propose the use of a combined protective culture containing selected strains of *P. chrysogenum* in combination with *Debaryomyces hansenii* as a promising strategy to reduce OTA production by *P. nordicum* in dry-cured hams. The efficacy of BCAs was tested in dry-cured hams under industrial ripening, resulting in a significant reduction of OTA contamination.

Three papers are focused on exploring phytochemical compounds as potential antifungal agents, two of them dealing with the use of garlic-derived compounds to control toxigenic molds in cereals and the other one with apple pomace. Quiles et al. [7] evaluate the antifungal activity of allyl isothiocyanate against aflatoxigenic *Aspergillus flavus* and ochratoxigenic *Penicillium verrucosum* on cereals in small-scale silos, obtaining a significant reduction of the *A. flavus* and *P. verrucosum* growth as well as an important reduction of the OTA. Mylona et al. [8] reported that the efficacy of treatment with propyl propane thiosulfonate and propyl propane thiosulfinate to reduce *Fusarium* growth and mycotoxin production was dependent on the specific “*Fusarium* species-toxin” pathosystem and the a_w_ of cereals. The work of Oleszek et al. [9] showed that apple pomace could be a good source of natural bio-fungicides inhibiting the growth of crop pathogens, mycotoxigenic molds, being the strongest antifungal activity exerted by a fraction containing phloridzin.

The use of mycotoxin-degrading methods is a promising approach to control this hazard and to counteract their toxic effects in livestock. With respect to enzymatic detoxification, Alberts et al. [10] developed an innovative method using the commercial fumonisin esterase FumD to reduce fumonisin B in whole maize, resulting in the formation of the hydrolyzed breakdown product, HFB1, associated with the aqueous phase to be discarded. Fruhauf et al. [11] concluded that the cleavage of ZEA by the zearalenone-lactonase Zhd101p reduces its estrogenicity in piglets, providing an important basis for the further evaluation of ZEA-degrading enzymes.

Finally, four papers explore the effectiveness of new technologies in preventing or reducing mycotoxin contamination of foods and feeds. Wang et al. [12] synthesized a light-responsive dendritic-like α-Fe_2_O_3_ that showed good activity for the photocatalytic degradation of deoxynivalenol (DON) in aqueous solution under visible light irradiation with a significant decrease of the toxicity of this mycotoxin. Casas-Junco et al. [13] propose a treatment of OTA-contaminated roasted coffee with cold plasma, achieving a decrease of OTA concentration and a reduction in toxicity of the treated coffee. Liu et al. [14] proved that the treatment of wheat kernels with superheated steam is an effective method to reduce the content of DON by thermal degradation, and the reduction rate increased significantly with the steam temperature. In addition, the treatment improves the qualities of crisp biscuits made from processed wheat. García-Díaz et al. [15] proposed a novel niosome-based encapsulated essential oil product applied to polypropylene bags that significantly reduced the development of *A. flavus* and aflatoxin contamination in maize up to 75 days. Thus, the correct application of this product may be a sustainable way to avoid the occurrence of aflatoxins in stored maize.

Therefore, the results contained in this Special Issue represent several interesting advances in different methodologies for the control of mycotoxins in food and feeds, providing useful information for the development of further research in this field.
